# Antibiotic Resistance and Sewage-Associated Marker Genes in Untreated Sewage and a River Characterized During Baseflow and Stormflow

**DOI:** 10.3389/fmicb.2021.632850

**Published:** 2021-06-11

**Authors:** Warish Ahmed, Pradip Gyawali, Kerry A. Hamilton, Sayalee Joshi, David Aster, Erica Donner, Stuart L. Simpson, Erin M. Symonds

**Affiliations:** ^1^CSIRO Land and Water, Ecosciences Precinct, Dutton Park, QLD, Australia; ^2^Institute of Environmental Science and Research Ltd. (ESR), Porirua, New Zealand; ^3^School of Sustainable Engineering and the Built Environment, Arizona State University, Tempe, AZ, United States; ^4^Biodesign Center for Environmental Health Engineering, The Biodesign Institute, Arizona State University, Tempe, AZ, United States; ^5^Department of Agriculture and Fisheries, Ecosciences Precinct, Dutton Park, QLD, Australia; ^6^Future Industries Institute, University of South Australia, University Boulevard, Mawson Lakes, SA, Australia; ^7^CSIRO Land and Water, Lucas Heights, NSW, Australia; ^8^College of Marine Science, University of South Florida, St. Petersburg, St. Petersburg, FL, United States

**Keywords:** microbial source tracking, antibiotic resistance, stormwater, human health risks, sewage pollution

## Abstract

Since sewage is a hotspot for antibiotic resistance genes (ARGs), the identification of ARGs in environmental waters impacted by sewage, and their correlation to fecal indicators, is necessary to implement management strategies. In this study, sewage treatment plant (STP) influent samples were collected and analyzed using quantitative polymerase chain reaction (qPCR) to investigate the abundance and correlations between sewage-associated markers (i.e., *Bacteroides* HF183, *Lachnospiraceae* Lachno3, crAssphage) and ARGs indicating resistance to nine antibiotics (belonging to aminoglycosides, beta-lactams, sulfonamides, macrolides, and tetracyclines). All ARGs, except *bla*_VIM_, and sewage-associated marker genes were always detected in untreated sewage, and *ermF* and *sul1* were detected in the greatest abundances. *intl1* was also highly abundant in untreated sewage samples. Significant correlations were identified between sewage-associated marker genes, ARGs and the *intl1* in untreated sewage (τ = 0.488, *p* = 0.0125). Of the three sewage-associated marker genes, the BIO-ENV procedure identified that HF183 alone best maximized correlations to ARGs and *intl1* (τ = 0.590). Additionally, grab samples were collected from peri-urban and urban sites along the Brisbane River system during base and stormflow conditions, and analyzed for *Escherichia coli*, ARGs, the *intl1*, and sewage-associated marker genes using quantitative polymerase chain reaction (qPCR). Significant correlations were identified between *E. coli*, ARGs, and *intl1* (τ = 0.0893, *p* = 0.0032), as well as with sewage-associated marker genes in water samples from the Brisbane River system (τ = 0.3229, *p* = 0.0001). Of the sewage-associated marker genes and *E. coli*, the BIO-ENV procedure identified that crAssphage alone maximized correlations with ARGs and *intl1* in river samples (τ = 0.4148). Significant differences in *E. coli*, ARGs, *intl1*, and sewage-associated marker genes, and by flow condition (i.e., base vs. storm), and site types (peri-urban vs. urban) combined were identified (*R* = 0.3668, *p* = 0.0001), where percent dissimilarities between the multi-factorial groups ranged between 20.8 and 11.2%. Results from this study suggest increased levels of certain ARGs and sewage-associated marker genes in stormflow river water samples compared to base flow conditions. *E. coli*, HF183 and crAssphage may serve as potential indicators of sewage-derived ARGs under stormflow conditions, and this merits further investigation. Data presented in this study will be valuable to water quality managers to understand the links between sewage pollution and ARGs in urban environments.

## Introduction

Sewage treatment plants (STPs) collect sewage from various sources, including households, hospitals, commercial and industrial sites followed by treatment processes to remove biological and chemical contaminants before the treated water is discharged back into the environment or recycled for commercial enterprises. Human and animal fecal waste contamination is a global problem and can occur from wet/dry weather overflows, septic tanks, faulty sewer lines, illicit sewer connections, lift stations, and in the event of natural disasters such as earthquakes and flooding (Olds et al., 2018; Ahmed et al., 2019a). Sewage contamination results in the dissemination of pathogens, nutrients, toxicants, endocrine disruptors, antibiotic resistant bacteria (ARB) and antibiotic resistance genes (ARGs) into the environment (Rodriguez-Manzano et al., 2010). In addition to sewage, defecation from wildlife, livestock, and pets contribute significant loads of pathogens, ARB, and ARGs to waterways by direct deposition or by stormwater runoff (Cox et al., 2005; Ahmed et al., 2019c).

Antibiotic resistant bacteria pose significant human health risks. For example, at least 2.8 million people contract an antibiotic-resistant infection, and more than 35,000 deaths occur each year in the United States as per CDC’s 2019 AR Threats Report (CDC, 2019). It has been estimated that antibiotic-resistant infections would be responsible for 10 million excess deaths globally, and a cumulative cost of US$ 100 trillion by 2050 if the current rapid evolution and spread of antimicrobial resistance are not abated (The Review on Antimicrobial Resistance, 2014). In Australia, 290–1,600 deaths/year are due to antibiotic resistance (Australian Commission on Safety and Quality in Health Care (ACSQHC), 2019). ARGs are considered emerging contaminants in the aquatic environments since they can be transferred to pathogenic bacteria in ecosystems via horizontal gene transfer (HGT) (Nguyen et al., 2019). Therefore, mitigation strategies are needed to prevent their widespread dissemination. The World Health Organization (WHO) identified water safety and improved sanitation as critical components in preventing the spread of ARB (WHO, 2014).

The presence of fecal contamination is typically determined by monitoring fecal indicator bacteria (FIB), such as *Escherichia coli* for freshwater and enterococci for marine and estuarine waters (Rochelle-Newall et al., 2015; Ahmed et al., 2019a). However, limitations of FIB monitoring include factors such as differential decay rates and poor correlations to pathogens in aquatic environments (Harwood et al., 2005; Signor et al., 2005; Wade et al., 2008; Ferguson et al., 2012; Korajkic et al., 2019), potential environmental, non-fecal sources (Badgley et al., 2011; Byappanahalli et al., 2012), and most importantly, their inability to identify the source of contamination (Harwood et al., 2014). Without knowing the sources, relative pathogen contributions cannot be assigned, thus inhibiting the ability to manage human health risks (Soller et al., 2015).

The application of microbial source tracking (MST) tools enabled researchers and regulators to differentiate between sources of fecal contamination (Harwood et al., 2014; Ahmed et al., 2016). The most widely used MST tools involve the analysis of host-associated marker genes using qPCR (Harwood et al., 2014). The benefit of host-associated marker genes is that they occur in far greater abundances compared to pathogens and are highly specific to the type of contamination source (e.g., human vs. wildlife) (Stoeckel and Harwood, 2008; Ahmed et al., 2016). These tools are currently being applied throughout the world to gain insight into the sources of fecal contamination in water bodies and to help guide risk management solutions (Harwood et al., 2014; Derx et al., 2016; Ahmed et al., 2018).

Sewage treatment plants are considered hot spots for ARGs (Rizzo et al., 2013; Pazda et al., 2019). Sewage-associated marker genes such as *Bacteroides* HF183, crAssphage, *Lachnospiraceae* Lachno3, and others are highly abundant in untreated sewage (Ahmed et al., 2016; Feng et al., 2018; Farkas et al., 2019). Therefore, in the event of recent sewage pollution, it is highly likely that both sewage-associated markers and certain ARGs will be present in environmental waters. A recent study analyzed relative ARG abundance and accompanying extent of fecal contamination in publicly available metagenomic data, using crAssphage sequences as a marker of sewage contamination (Karkman et al., 2019). The authors were investigating whether an increased abundance of ARGs in sewage and sewage-impacted environments was due to on-site selection pressure by residual antibiotics or was simply a result of fecal pollution with ARB. The analysis suggested that the presence of ARGs in the environments can largely be explained by fecal pollution, with no clear signs of on-site selection in the environment.

Patterns of increased human fecal pollution and ARGs were previously identified in environmental waters (Ahmed et al., 2018; Reynolds et al., 2020; Stange and Tiehm, 2020; Moretto et al., 2022). Abundances of 47 ARGs in several storm drain outfalls during dry and wet weather in Tampa Bay, FL, United States along with sewage-associated *Bacteroides* HF183 and crAssphage markers were monitored (Ahmed et al., 2018). The study found that the abundances of sewage-associated markers and many ARGs in were relatively high, in water samples collected during wet weather compared to dry weather, and that storm drain outfalls may be potential hot spots for microbial contamination in Tampa Bay. CrAssphage and HF183 were significantly correlated with *intl1*, and several ARGs such as *sul1*, tet*(M)*, *ampC*, *mexB*, and tet*(W)*. Similarly, greater abundances of human MST markers and ARGs were measured after heavy rains in a German spring (Stange and Tiehm, 2020). While HF183 was consistently and significantly correlated to several ARGs in the Liffey Estuary, Ireland, HF183 was not always significantly correlated with ARGs in the urban streams nearby (Reynolds et al., 2020).

Information on the positive correlation between the presence of sewage-associated marker genes and ARGs in environmental waters may aid in the management of fecal pollution and ARG dissemination. Yet it is also possible that ARG abundance may be linked to animal fecal (Lee et al., 2020) and non-fecal sources. Sediments can potentially act as reservoirs for ARB and ARG, and resuspension from sediments during storm events or due to other disturbances, is likely to occur. Furthermore, ARGs have also been detected in Lake Tai, China, despite low levels of human, ruminant, and pig fecal pollution (Stange et al., 2019).

The main objective of this study was to better understand the abundance and correlation between sewage-associated markers and ARGs in sewage and river water exposed to variable amounts of fecal pollution. To achieve this objective, first we investigated the abundance and correlations between ARGs and sewage-associated marker genes in untreated sewage. Subsequently we characterized the occurrence and abundance of *E. coli*, ARGs, and sewage-associated marker genes at a range of peri-urban to urban sites along the Brisbane River system, which were exposed to different amounts of fecal pollution. Correlations between *E. coli*, ARGs, and sewage-associated marker genes in a river system were analyzed to identify if any sewage indicator could be used to imply the presence of ARGs in river water. Finally, we compared the occurrence and abundance of *E. coli*, ARGs, and sewage-associated marker genes in water samples collected during baseflow and under stormflow conditions to better understand the dynamics between them. This study is the first of its kind to elucidate the relation between sewage-associated marker genes and ARGs in an Australian urban river system.

## Materials and Methods

### Untreated Sewage Sampling

Untreated sewage samples were collected from a municipal STP in Southeast Queensland (SEQ), Australia. The STP treats sewage from approximately 250,000 people and a hospital. The treatment process consists of primary treatment, a secondary treatment (activated sludge), and disinfection with chlorine and UV. Approximately 100 mL of untreated sewage (grab) samples were collected in sterile bottles from the influent of the STP. Weekly samples were collected in triplicate over a period of 6 weeks yielding 18 samples in total for monitoring of ARGs, and sewage-associated marker genes. Samples were transported on ice to the laboratory and stored at 4°C for up to 24 h before analysis.

### Environmental Water Sampling

**FIGURE 1:**
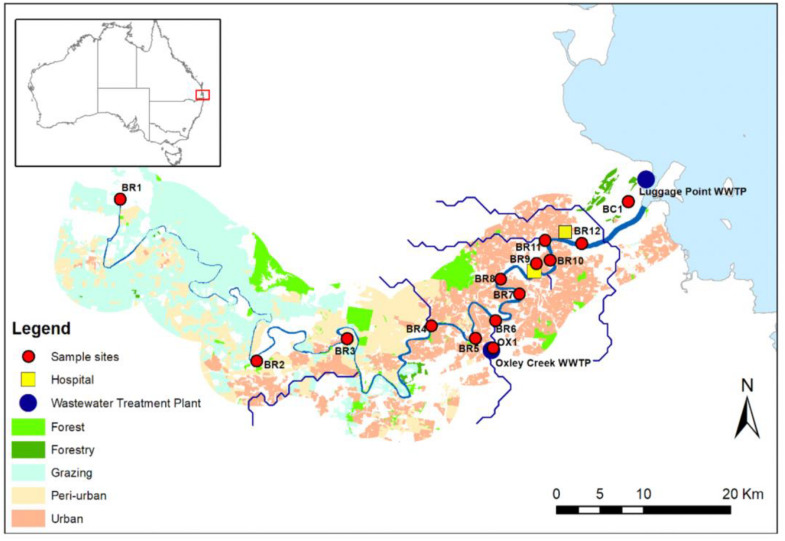
Map of the study sites along the Brisbane River system and its tributaries Oxley Creek, and Boggy Creek, located in Brisbane, Australia. Site OX1 is located downstream of a WWTP, site BR8 is located in proximity to a storm water drain, and sites BR9, BR10, BR11, and BR12 are located downstream of hospitals.

Water samples (i.e., single grab) were collected from Brisbane River and associated creeks (Oxley Creek and Boggy Creek) in Brisbane, Australia (Figure 1). Oxley Creek is a tributary of Brisbane River and is tidally influenced. Boggy Creek is a tributary of lower Brisbane River and drains into the mouth of Brisbane River. Twelve sampling sites (BR1-BR12) were chosen along the entire length (i.e., 344 km) of the river. In addition, one sampling site (OX1) was chosen in Oxley Creek and one sampling site (BC1) was chosen in Boggy Creek. Sampling sites BR1-BR5 are sparsely populated with forested hills and grazing land. Based on land-uses and population, these sites are considered peri-urban sites. The middle and lower catchments (sites BR6-BR12, OX1, and BC1) are highly populated and characterized by industrial, residential, and urban areas. From each site, samples were collected on four separate occasions between 15 and 26th May [15th May (Event 1), 19th May (Event 2), and 26th May (Event 3)], 2019 and 13th February (Event 4) 2020. Samples collected between 15 and 26th May represented baseflow samples when the study area did not receive any precipitation 10 days before the respective water sampling. Samples collected on the 13th of February 2020 represented a storm weather event when the study area received 78.8 mm rainfall during the 3 days prior to sampling. The study area did not receive any additional rainfall to allow more sampling to be undertaken during stormflow conditions. Baseflow samples were collected during low tides, while stormflow samples were collected during a spring tide. A total of 56 water samples were collected for monitoring of *E. coli*, ARGs, and sewage-associated marker genes. For river water samples, *E. coli* was included as a general FIB to indicate fecal pollution from both humans and animals. Water samples were collected in 500 mL sterilized PET bottles at approximately 30 cm below the water surface and transported on ice to the laboratory and analyzed within 16 h.

### Concentration of River Water Samples

For qPCR analysis of *E. coli*, ARGs, and sewage-associated marker genes, 500 mL of each water sample was filtered through a 90-mm, 0.45-μm pore size HAWP membrane (Millipore, Tokyo, Japan). Before filtration, the pH in all water samples was adjusted to 3.5, using 2.0 N HCl to capture both bacteria and viruses simultaneously (Ahmed et al., 2015).

### DNA Extraction

DNA was extracted from an aliquot of 250 μL of untreated sewage sample using the MO Bio PowerSoil DNA isolation kit (Mo Bio Laboratories, Carlsbad, CA, United States) with minor modifications as described elsewhere (Ahmed et al., 2015). For the river water samples, a DNeasy PowerWater Kit (Qiagen, Valencia, CA, United States) was used to extract DNA directly from the membrane. DNA concentrations were measured with a spectrophotometer (NanoDrop ND-1000, Thermo Scientific, Wilmington, DE, United States). All DNA samples were stored at −80°C until further analysis.

### PCR Inhibition

An experiment was conducted to determine the presence of PCR inhibitors in DNA samples from untreated sewage and water samples collected from the Brisbane River system using a Sketa22 qPCR assay (Haugland et al., 2005). DNA samples with a 2-quantification cycle (Cq) delay were considered to have potential PCR inhibitors (Ahmed et al., 2019a). Samples with PCR inhibitors were subjected to a 10-fold dilution with TE buffer and reanalyzed with the Sketa22 assay. PCR-uninhibited and 10-fold diluted (inhibition relieved) samples were used for qPCR analysis.

### qPCR Assays

Previously published qPCR assays were used for the analysis of *E. coli* 23S rRNA gene (Chern et al., 2011), *aacA* (Szczepanowski et al., 2009), *bla*_ctx–m–__32_ (Szczepanowski et al., 2009), *bla*_KPC_ (Hindiyeh et al., 2008), *bla*_VIM_ (Szczepanowski et al., 2009), *ermF* (Ma et al., 2011), *intl1* (Goldstein et al., 2001), *sul1* (Heuer and Smalla, 2007), *sul2* (Szczepanowski et al., 2009), *tet(M)* (Ng et al., 2001), and *vanA* (Bell et al., 1998), and sewage-associated marker genes HF183 (Green et al., 2014), crAssphage CPQ_056 (Stachler et al., 2017), and Lachno3 (Feng et al., 2018). For this study, we chose *E. coli* as it has been recommended that *E. coli* is suitable for freshwater monitoring by the National Health and Medical Research Council (NHMRC, 2008). For ARGs, we chose aminoglycosides, beta-lactams, sulfonamides, macrolides, and tetracycline resistance genes because of their widespread usage in animal farming worldwide. More than half of the antibiotics prescribed to humans are beta-lactams (Lachmayr et al., 2009; Al Salah et al., 2019). We chose *aacA*, *bla*_ctx–m–__32_, *bla*_KPC_, *bla*_VIM_, *ermF*, *sul1*, *sul2*, *tet(M)*, *vanA*, and the *intl1* because these were suggested as possible indicators to assess the antibiotic resistance status in environmental settings and some of them are highly prevalent in contaminated environments (Berendonk et al., 2015; Gillings, 2018). We also chose three sewage-associated marker genes namely HF183, Lachno3, and crAssphage to provide evidence of sewage contamination in the studied river system. These markers were selected based on their high host-specificity and sensitivity in Brisbane, Australia as determined in our previous studies (Hughes et al., 2017: Ahmed et al., 2019b). The primers and probes for each assay are shown in Supplementary Table 1 along with qPCR cycling parameters. All qPCR amplifications were performed in 20 μL reaction mixtures using SsoAdvanced Universal Probes Supermix or SsoAdvanced Universal SYBR Green Supermix (Bio-Rad Laboratories, Richmond, CA, United States).

For HF183, Lachno3, and CPQ_056 assays, qPCR mixtures contained 10 μL of SsoAdvanced Universal Probes Supermix, 1000 nM forward primer, 1000 nM reverse primer, and 100 nM of probe (for the HF183, Lachno3, and CPQ_056). For ARGs, qPCR mixtures contained 10 μL of SsoAdvanced Universal SYBR Green Supermix, 300 nM of the forward primer, and 300 nM of the reverse primer. To separate the specific product from non-specific products, including primer dimers, a melting curve analysis was performed for each qPCR run. During the melt curve analysis, the temperature was increased from 65 to 95°C at 0.5°C increment. Samples were considered positive when the melting points were matched with the melting point of the standard curve amplification within a tolerance of 0.5°C (Nutz et al., 2011).

The qPCR assays were performed using a Bio-Rad CFX96 thermal cycler. All qPCR reactions were performed in triplicate. gBlocks gene fragments were used to prepare qPCR standards, ranging from 10^6^ to 1 gene copies (GC)/μL of DNA (Integrated DNA Technology, Coralville, IA, United States). For each qPCR run, a series of standards (3 × 10^6^ to 3 GC/reaction), and no template controls (*n* = 3) were included. A standard curve was generated for each assay and instrument run.

Quantitative polymerase chain reaction performance characteristics such as amplification efficiencies (E), correlation coefficient (r^2^), slopes and Y-intercepts were determined from the standard curves for each assay and were within the prescribed limits (Bustin et al., 2009). The assay limit of detection (ALOD) and quantification (ALOQ) for the different assays used were defined as the minimum copy number detected (e.g., lowest copy number detected 95% of the time) and quantifiable (e.g., lowest copy number detected 100% of the time), respectively, as previously described (Verbyla et al., 2016). The abundances in the original sample were back-calculated to take into account each step of the methods. Any sample with triplicate Cq measurements below the ALOQ was considered detected but not quantifiable.

### Quality Control

A reagent blank was included for each batch of DNA samples to ensure no carryover contamination occurred from DNA extraction reagents. No carryover contamination was observed in extracted DNA samples. To minimize qPCR contamination, DNA extraction and qPCR setup were performed in separate laboratories.

### Data Analysis

All descriptive and multivariate statistical analyses were executed in R version 4.0.2 using the NADA and Vegan packages (R Core Team, 2013; Oksanen et al., 2016; Lee, 2017). All data were interval censored, differentiating between non-censored (i.e., quantifiable) measurements, and left-censored (i.e., concentrations less than the ALOD, and those positive, but not quantifiable), following the recommendations for left-censored data (Helsel, 2011). To execute multivariate statistical analyses that accommodate left-censored data, the u-Score ranks were calculated from the log_10_ transformed data (https://practicalstats.teachable.com/ on June 20, 2020) (Helsel, 2011, 2019). Then Euclidean distance matrices were calculated from the u-Score ranks (Helsel, 2011) prior to analyses.

### Descriptive Statistics

With respect to the sewage samples, the mean concentration of each microbial target was calculated from the triplicate samples collected during each sampling event (*n* = 6) and used in subsequent analyses. The mean, standard deviation, and frequency of detection were determined for each microbial target in sewage. For all river water samples, the median, minimum, and maximum were reported. For microbial targets with censored observations (up to 80% censored), the median was estimated using Robust Regression on Order Statistics (rROS) (lognormal distribution assumed for modeling the left-censored portion of the distribution). If more than 80% of observations were censored, then only the minimum and/or maximum values measured were reported taking into account the process limit of detection (PLOD) and process limit of quantification (PLOQ). PLOD and PLOQ were calculated from ALOD and ALOQ values.

### Multivariate Correlation Analyses

To understand correlations between sewage-associated marker genes and ARGs in sewage at the community-composition-level, the Mantel test (multivariate approach) was executed using the Kendall method and 9,999 permutations as recommended (*n* = 6 per microbial target; Legendre and Legendre, 2012). Subsequently, the best subset of sewage-associated markers that maximized the correlations with the ARGs was identified using the BIO-ENV procedure [iterative Mantel tests with the Kendall method designed to identify the best subset of a group of variables, such that they maximize (rank) correlations with a dissimilarity matrix of other variables; Clarke and Ainsworth (1993)]. Similarly, the Mantel test and BIO-ENV procedure were also used to identify correlations between *E. coli*, sewage-associated marker genes, and ARGs at the community-composition-level in the river water (*n* = 56 per microbial target).

### Multivariate Hypothesis Testing

Analysis of similarities (ANOSIM) with 9,999 permutations was executed to test whether there was a significant difference in *E. coli*, sewage-associated markers, and ARG abundances in the river between samples collected from different site types [urban (*n* = 9 sites) vs. peri-urban (*n* = 5 sites)] and flow conditions [baseflow (*n* = 42 samples) vs. stormflow (*n* = 14 samples)], separately, and combined [urban baseflow (*n* = 27 samples), urban stormflow (*n* = 9 samples), peri-urban baseflow (*n* = 15 samples), peri-urban-stormflow (*n* = 5 samples)] (Clarke, 1993). The Similarity Percentages (SIMPER) procedure was executed with 9,999 permutations to determine which microbial targets differed between groupings of site type and stormflow. Non-metric multidimensional scaling (NMDS) was also used to visualize differences between site types and flow conditions. A *p*-value of <0.05 was considered significant for all multivariate analyses.

## Results

### Prevalence and Abundance (log_10_ Copies/L) of ARGs and Sewage-Associated Marker Genes in Untreated Sewage Samples

**FIGURE 2:**
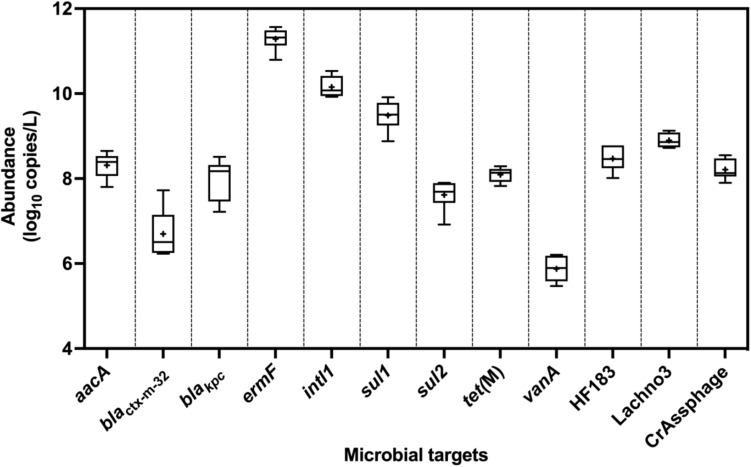
Abundance (log_10_ copies/L) of ARGs (*aacA*, *bla*_ctx–m–__32_, *bla*_KPC_, *ermF*, *sul1*, *sul2*, *tet(M)*, and *vanA*), *intl1*, and sewage-associated marker genes (HF183, crAssphage CPQ_056, and Lachno3) in untreated sewage samples, which were always detected in quantifiable abundances. ARG *bla*_VIM_ was positively detected, but not in quantifiable abundances (between 2.42- and 2.59-log_10_ copies/L), in only two of the six untreated sewage samples. +denotes mean while the outer box lines represent 25th and 75th percentiles and the whiskers extended to the range, and lines inside the boxes represent median values.

All ARGs and sewage-associated marker genes were detected in samples collected from six out of the six sampling events except *bla*_VIM_, which was only detected twice out of the six sampling events but in abundances less than the ALOQ (Figure 2). Among the ARGs tested, the mean abundances of *ermF* (11 ± 0.27 log_10_ copies/L) and *sul1* (9.50 ± 0.25 log_10_ copies/L) were ∼1.10 to 5.50 log_10_ copies greater than *aacA*, *bla*_KPC_, *bla*_ctx–m–__32_, *sul2*, *tet(M)*, and *vanA*. The mean abundance of *intl1* was 10 ± 0.25 log_10_ copies/L of sewage. Among the three sewage-associated marker genes, the mean abundance (8.90 ± 0.17 log_10_ copies/L) of the Lachno3 was ∼0.40 to 0.70 log_10_ greater than HF183 (8.50 ± 0.30 log_10_ copies/L) and crAssphage (8.20 ± 0.24 log_10_ copies/L).

### Occurrence and Abundance of *E. coli*, ARGs, and Sewage-Associated Marker Genes in Water Samples From the Brisbane River System

A total of 56 river water samples were collected from sampling 14 sites on four occasions, in which three occurred during baseflow conditions and one occurred during stormflow conditions. All water samples collected during the baseflow and stormflow from peri-urban and urban sites were positive for *E. coli*. The ARGs *bla*_ctx–m–__32_, *sul2*, and *vanA* were not detected in any water sample collected during baseflow from these peri-urban and urban sites. *intl1* and ARG *sul1* were most frequently detected in both baseflow and stormflow, however, the frequency of detection was greater in peri-urban sites compared to urban sites. The occurrence of *aacA*, *bla*_VIM_, *ermF*, *sul1*, *sul2*, *tet(M)*, and *intl1* and all three sewage-associated marker genes was greater in stormflow than baseflow samples for both peri-urban and urban sites.

Among the 14 sites [five peri-urban (BR1-BR5) and nine urban sites (BR6-BR12, OX1, and BC1)] sampled in baseflow at Event 1, water samples from upstream peri-urban sites (i.e., BR1, BR2, and BR3) and sites located near the STP or stormwater drains (OX1, BR8, and BR12) were positive for one to three ARGs and *intl1* but negative (i.e., <ALOD) for sewage-associated marker genes (Supplementary Table 2). Similar patterns were also observed for the samples collected during the Event 2, however, the sample from site BR8 was positive for four ARGs and three sewage-associated marker genes. Sewage-associated marker genes could not be detected in all water samples collected during baseflow at Event 3; however, water samples from upstream sites (i.e., BR1, BR2, and BR3) and one urban site (i.e., OX1) located downstream of the STP were positive for two to four ARGs. Most of the samples collected during stormflow at Event 4 were positive for multiple ARGs and sewage-associated marker genes except for a few urban sites (i.e., BR7, BR9, BR10, and BR11). Notably, the sample collected from site BC1 was heavily polluted (i.e., positive for ten ARGs and three sewage-associated marker genes).

**FIGURE 3:**
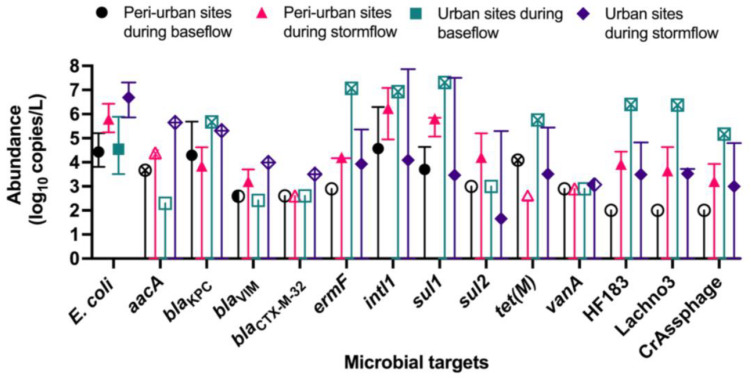
Median abundance (log_10_ copies/L) with maximum and minimum abundances (error bars) reported for *E. coli* (PLOD = 2.00 log_10_ copies/L), *aacA* (PLOD = 2.30 log_10_ copies/L), *bla*_ctx–m–__32_ (PLOD = 2.60 log_10_ copies/L), *bla*_KPC_ (PLOD = 2.30 log_10_ copies/L), *bla*_VIM_ (PLOD = 2.42 log_10_ copies/L), *ermF* (PLOD = 2.90 log_10_ copies/L), *intl1* (PLOD = 2.30 log_10_ copies/L), *sul1* (PLOD = 2.00 log_10_ copies/L), *sul2* (PLOD = 3.00 log_10_ copies/L), *tet*(M) (PLOD = 2.60 log_10_ copies/L), and *vanA* (PLOD = 2.90 log_10_ copies/L), and sewage-associated marker genes (HF183, crAssphage CPQ_056, and Lachno3; all PLOD = 2.00 log_10_ copies/L) in water samples collected from peri-urban and urban sites from the Brisbane River system during the base and stormflow (solid-filled shape). If a particular microbial target had left-censored values, the uncertainty of the minimum abundance was depicted with a straight, vertical line. If it was not possible to calculate a median abundance (>80% left-censored), then the maximum abundance measured was depicted with an “X” inside the site/flow shape. When a microbial target was only detected below the limit of quantification, then a half-filled shape depicted the limit of quantification. Finally, an un-filled shape represents 100% left-censored values, and the process limit of detection is depicted.

The abundances of *E. coli*, ARGs, and sewage-associated marker genes are shown in Figure 3. The levels of *E. coli* in water samples collected during the baseflow from peri-urban sites ranged from 3.80- to 5.20-log_10_ copies/L with a median value of 4.43-log_10_ copies/L. The levels of *E. coli* in urban sites baseflow samples were slightly greater than in peri-urban sites. We observed increased levels (i.e., ∼2–3 orders of magnitude greater) of *E. coli* in stormflow samples collected from both urban (range = 5.86- to 7.30-log_10_ copies/L with a median value of 6.60-log_10_ copies/L) and peri-urban (range = 5.24- to 6.42-log_10_ copies/L with a median value of 5.79-log_10_ copies/L) sites compared to baseflow samples.

The abundance of *bla*_Kpc_ in baseflow water samples ranged from 2.47- to 5.69-log_10_ copies/L with a median value of 4.29-log_10_ copies/L for peri-urban sites; however, *bla*_Kpc_ was rarely detected in urban sites and when detected, abundance ranged from 2.47- to 5.69-log_10_ copies/L. Other ARGs such as *aacA* (range 2.47- to 5.65-log_10_ copies/L), *bla*_VIM_, (range 2.59- to 3.99-log_10_ copies/L) *bla*_CTX–M–__32_ (2.77- to 3.50-log_10_ copies/L), and *ermF* (range 2.90- to 7.07-log_10_ copies/L) were sporadically detected in baseflow and stormflow samples in both urban and peri-urban sites. The abundance of *intl1* (range 4.95- to 7.86-log_10_ copies/L) and *sul1* (5.85 ± 7.50-log_10_ copies/L) were generally greater in stormflow samples collected from both urban and peri-urban sites compared to the baseflow events (2.30- to 6.93-log_10_ copies/L for *intl1* and 2.00- to 7.31-log_10_ copies/L for *sul1*). The abundances of *sul2* in water samples collected from urban and peri-urban sites during the stormflow ranged from 3.00- to 5.30-log_10_ copies/L, while *sul2* could not be detected (i.e., <PLOD) in any water samples collected during the baseflow conditions. Among the three sewage associated marker genes, the abundance of Lachno3, HF183, and crAssphage in stormflow samples ranged from 2.17- to 4. 69-, 2.17- to 4. 83-, and 2.17- to 4.80-log_10_ copies/L, respectively, and were detected in both urban and peri-urban. In contrast, these marker genes were not detected in baseflow water samples collected from peri-urban sites but were present in an urban site (BR8; Event 2).

### Correlations at the Community-Composition-Level Between Microbial Targets in Untreated Sewage and Water Samples From the Brisbane River System

Significant correlations at the community-composition-level were identified between sewage-associated markers and ARGs in untreated sewage samples (*n* = *6*) using the Mantel test (τ = 0.488, *p* = 0.0125). Among all of the sewage-associated markers, the BIO-ENV procedure identified that HF183 alone best maximized correlations to ARGs in untreated sewage samples (τ = 0.5898). Significant correlations at the community-composition-level were also identified between ARGs and *E. coli* (τ = 0.0893, *p* = 0.0032), as well as with sewage-associated markers in the 56 river water samples (τ = 0.3229, *p* = 0.0001), separately. Of *E. coli* and the sewage-associated markers combined, the BIO-ENV procedure identified that crAssphage alone maximized correlations with patterns of ARGs in the river water samples (τ = 0.4148).

### Differences in *E. coli*, Sewage-Associated Markers, and ARGs Observed by Flow Conditions and Site Types in Water Samples From the Brisbane River System

**FIGURE 4:**
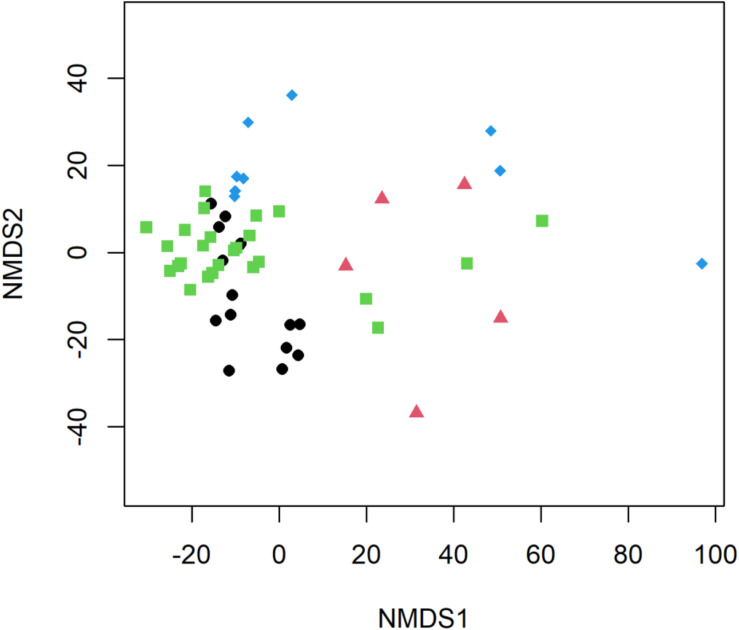
Non-metric multidimensional scaling (NMDS) plot of *E. coli*, ARGs, and sewage-associated markers, measured in water samples (*n* = 56) collected from peri-urban sites during baseflow (black circle) and stormflow (red triangle), as well as and urban sites during baseflow (green square) and stormflow (blue diamond) along the Brisbane River system, Brisbane, Australia.

Significant differences in *E. coli*, ARGs, *intl1*, and sewage-associated markers occurred under different flow conditions [baseflow (*n* = 42 samples) vs. stormflow (*n* = 14 samples); *R* = 0.5340, *p* = 0.0001], while non-significant weak differences were identified by site types [urban (*n* = 36 samples) vs. peri-urban (*n* = 20 samples); *R* = 0.09823, *p* = 0.0529]. Subsequently, differences in *E. coli*, ARGs, and sewage-associated markers in river water samples were tested using a multi-factorial ANOSIM analysis and significant differences were identified by stormflow and site type combined [urban baseflow (*n* = 27 samples), urban stormflow (*n* = 9 samples), peri-urban baseflow (*n* = 15 samples), peri-urban-stormflow (*n* = 5 samples); *R* = 0.3668, *p* = 0.0001], which were also visualized in the NMDS plot (Figure 4). SIMPER analysis revealed that average percent dissimilarities were greatest between urban-baseflow vs. peri-urban-stormflow (20.8%), followed by peri-urban-stormflow vs. baseflow (19.5%), urban-stormflow and peri-urban baseflow (19.3%), urban- vs. peri-urban during stormflow (18.9%), urban-baseflow vs. -stormflow (17.6%), and urban vs. peri-urban during baseflow (11.2%) (Table 1). Depending upon site type-flow condition groupings, four to eight microbial targets were identified as the most influential in contributing to the differences observed between multi-factorial groupings. Only *bla*_KPC_ and *intl1* significantly differed when comparing peri-urban and urban sites during baseflow conditions. The microbial targets that most frequently were identified as most influential with respect to the differences observed by site type and flow condition included *E. coli*, two sewage-associated markers (HF183 and crAssphage), as well as *sul1* and *intl1*.

**TABLE 1:** The average percent dissimilarities and the corresponding percent contributions of the most influential (explaining >70% of the differences) microbial targets (*E. coli*, sewage-associated markers, and ARGs) observed between the multi-factorial groupings (flow condition and site type) for river water samples, as identified by the Similarity Percentages (SIMPER) procedure.

Multifactorial Grouping	Urban Sites in Baseflow conditions		Urban Sites in Stormflow conditions		Peri-urban Sites in Baseflow conditions	
	Average dissimilarity (%)	Contributions of the most influential microbial targets (%)	Average dissimilarity (%)	Contributions of the most influential microbial targets (%)	Average dissimilarity (%)	Contributions of the most influential microbial targets (%)
**Urban Sites in Stormflow conditions**	17.6%	*E. coli* (20.5%)				
		*sul1* (10.4%)*				
		*ermF* (9.6%)				
		*intl1* (9.4%)*				
		*tet(M)* (9.0%)				
		HF183 (8.5%)				
		CrAssphage (8.2%)				
**Peri-urban Sites in Baseflow conditions**	11.2%	*intl1* (19.4%)	19.3%	*E. coli* (19.6%)		
		*sul1* (18.0%)*		*intl1* (11%)*		
		*E. coli* (16.9%)*		*sul1* (10.9%)*		
		*bla*_KPC_ (16.2%)		*bla*_KPC_ (8.3%)*		
				*ermF* (8.4%)		
				*tet(M)* (7.7%)*		
				HF183 (7.2%)*		
**Peri-urban Sites in Stormflow conditions**	20.8%	*sul1* (14.2%)	18.9%	*sul1* (14.5%)	19.5%	*E. coli* (14.2%)*
		*intl1* (13.6%)		*intl1* (13.7%)		*sul2* (13.0%)
		*E. coli* (12.4%)*		*sul2* (10.0%)		*sul1* (11.5%)*
		*sul2* (12.3%)*		HF183 (8.5%)*		*intl1* (9.6%)*
		HF183 (8.7%)		CrAssphage (8.1%)		HF183 (9.5%)
		CrAssphage (8.4%)		*ermF* (7.7%)*		CrAssphage (9.0%)
		Lachno3 (7.0%)*		Lachno3 (7.4%)*		*bla*_VIM_ (7.9%)
				*tet(M)* (6.4%)*		

For each microbial target identified among the most influential, all percent contributions had *p* values (*p* < 0.05, unless indicated by an asterisk (*).

## Discussion

Antibiotic resistance genes are considered emerging contaminants (Pruden et al., 2006). Additionally, they are considered causes of a potential hazard whether contained within an ARB or as naked DNA, due to the fact that genetic material can be transferred between bacteria through mechanisms of HGT (Thomas and Nielsen, 2005). Although a previous study reported the presence of class 1 integron integrase genes in *E. coli* isolates in water samples collected from the Brisbane River system (Sidhu et al., 2017), little is known regarding the occurrence of ARGs and sewage-associated marker genes in baseflow and stormflow water samples in Brisbane, Australia. We determined the abundances of several ARGs, *intl1*, and sewage-associated marker genes in untreated sewage samples and then determined their abundances in baseflow and stormflow samples collected from the Brisbane River system characterized by peri-urban and urban areas.

The abundance of *ermF* was the greatest in sewage samples followed by *intl1* and *sul1*. These genes are also reported to be highly prevalent in sewage in Europe (Cacace et al., 2019; Osiñska et al., 2020; Pärnänen et al., 2019). Schmitz et al. (2019) reported the co-occurrence and high correlation between *intl1* and *sul1* throughout sewage treatment processes in the United States. The high abundances of *intl1* in untreated sewage samples are not surprising as human feces often carry up to 10^11^ copies of *intl1*/g of feces (Gillings, 2018).

Sewage samples were also positive for the carbapenemase encoding gene *bla*_VIM_, which is an emerging resistance in Australian human infections and has been implicated in a small number of outbreaks in Queensland (Australian Commission on Safety and Quality in Health Care (ACSQHC), 2019). Human infections associated with the ARG *bla*_KPC_ in Queensland are considered to be rare but the abundance was greater in sewage samples compared to *bla*_VIM_. The prevalence of emerging ARGs, such as *bla*_KPC_, *bla*_VIM_, and *bla*_ctx–m__–__32_, provides baseline data but are not yet prevalent in human infection monitoring data from Australia. It is likely that the abundance of these genes will be increased in sewage and also their abundance is expected to potentially increase in environmental waters.

The abundances of sewage-associated markers HF183, Lachno3, and crAssphage under stormflow conditions corroborates with previous studies and shows them to be highly sensitive marker genes for tracking sewage pollution in environmental waters (Hughes et al., 2017; Ahmed et al., 2019b; Korajkic et al., 2020). The host-specificity of sewage-associated markers were not determined in this study as in our previous studies, we extensively evaluated the host-specificity of the HF183, Lachno3, and crAssphage marker genes (Hughes et al., 2017; Ahmed et al., 2019b). Abundances of *ermF*, *intl1*, and *sul1* were greater in sewage compared to sewage-associated marker genes suggesting that in the event of sewage pollution in environmental waters, these ARGs are likely to be present. The concentrations of these three genes were greater in sewage compared to HF183 and Lachno3 probably due to the high antibiotic usage and associated gene transfer from bacteria to bacteria. *intl1* data presented in this study corroborate with a previous report that *intl1* may be a useful proxy for anthropogenic pollution in catchment waters (Borruso et al., 2016; Gillings, 2018).

Overall, significant differences in *E. coli*, sewage-associated markers, and ARGs were identified by site type and flow conditions (Figure 4). All water samples collected from the Brisbane River system in this study were positive for *E. coli* regardless of flow conditions and site types. We did not see a stark difference between the levels of HF183 and crAssphage in water samples collected between urban and peri-urban sites during the baseflow, suggesting human fecal pollution may not be occurring in many sites in the studied river system during baseflow conditions except an urban site (i.e., BR8) at event 2 (one of three base flow events), which was in close proximity to a storm drain. However, the abundance of *E. coli*, HF183, and crAssphage increased significantly in stormflow samples collected from both urban and peri-urban sites compared to baseflow samples. A previous study reported a greater abundance of *E. coli* and enteric pathogens in water samples collected from Brisbane River and its tributaries in stormflow compared to baseflow (Sidhu et al., 2012). Similarly, in North Queensland, higher abundances of *E. coli* were found near sewage outfalls, with a change in the microbial community noted between baseflow and stormflow water samples (Neave et al., 2014). For *E. coli* monitoring, we used qPCR, however, the guideline values are based on culturable FIB with the most stringent category “A” setting a limit at less than or equal to 40 CFU/100 mL. Therefore, *E. coli* was not comparable to this guideline. Nevertheless, the combined presence of *E. coli* and two sewage-associated markers indicated an increase in fecal pollution during storm conditions. *E. coli* was also detected in water samples that were negative for sewage-associated marker genes, suggesting that animal fecal pollution is also occurring in the Brisbane River system. The extraintestinal growth of *E. coli* in the Brisbane River system also cannot be ruled out and requires further investigation by characterizing *E. coli* or applying animal fecal markers to determine the sources of *E. coli*.

When comparing the different combinations of site types and flow conditions, different ARGs were identified as the most influential depending upon the site type and flow condition. *intl1* and *sul1* were always identified as contributing most to differences observed regardless of the groupings, followed by *sul2*, *tet(M)*, and *ermF*. The abundance of *intl1* and *sul1* were generally greater in stormflow samples collected from both urban and peri-urban sites, compared to the baseflow samples from the same sites suggesting surface run-off from point and non-point sources contribute ARGs to the Brisbane River system.

While HF183 and crAssphage significantly contributed to the differences observed for each site type when comparing flow conditions, the occurrence and abundance of all three sewage-associated marker genes were greater in stormflow samples compared to baseflow water samples for both urban and peri-urban sites. With differential persistence of bacteria and the potential for HGT, stormflow conditions may result in increased proliferation of ARGs. The presence of sewage-associated marker genes also indicates the potential presence of enteric viruses in the Brisbane River system (Sidhu et al., 2012). These factors collectively may increase health risks to recreational users.

As there are numerous ARGs that can be present in the environment with relevance to public health (Gatica et al., 2016), at sometimes high background levels can be detected even in “pristine” environments, there is not yet a risk-based consensus regarding which ARGs would be “high priority” for monitoring purposes (Hamilton et al., 2020). Therefore, assessing the correlation of ARGs with more commonly monitored factors provides value if surrogate monitoring targets could reasonably be used for assessing the antibiotic resistance impacts of stormwater on receiving water bodies. ARGs were significantly correlated with sewage-associated markers and *E. coli*. CrAssphage alone maximized correlations to the group of ARGs analyzed in river water samples collected from peri-urban and urban areas. Interestingly, HF183 alone best maximized correlations to ARGs in untreated sewage.

Different sewage-associated markers were identified to maximize correlations to ARGs depending on the matrix, thus emphasizing the importance of assessing correlations in the matrix of interest as ARG and sewage-associated marker persistence likely varies. Previous studies noted positive correlations between FIB and antimicrobial resistance in impacted surface waters in Southeast Queensland, indicating their role as a potential reservoir of resistance (Watkinson et al., 2007). Stachler et al. (2019) reported greater abundances of ARGs and crAssphage on wet weather days than on dry weather days. They also demonstrated a strong correlation of various ARGs with culturable *E. coli*, culturable enterococci, and HF183 qPCR assay, suggesting that the bacterial-based markers could be an indicator for the presence of ARGs in environmental waters due to human fecal pollution. Nevertheless, the results presented here demonstrate the application of sewage-associated marker genes and *E. coli* as indicators of probable ARG prevalence under stormflow conditions but requires further validation. The other benefits of the sewage-associated marker genes are that their presence can be used to identify hot spots of sewage pollution or to detect broken pipes. This could be a useful source control option to decrease environmental spread of ARGs.

In this study, sewage-associated marker genes were detected near the storm drains such as site BR8 (Event 2). Interestingly, most of the samples collected during stormflow (Event 4) were positive for multiple ARGs and sewage-associated marker genes, suggesting various point and non-point sources are contributing to these microbial targets. Notably, the sample collected from site BC1 (urban) was positive for ten ARGs and three sewage-associated marker genes. Site BC1 is located downstream of an urban WWTP. The water samples from this site and others in the upstream sites were collected during the high tide. It is highly likely that high tide pushed ARGs and sewage-associated marker genes upstream to site BR12. We also noted increased levels of *ermF*, *intl1*, and *sul1* at sites BR5, OX1, BR12, and BC1 in the stormflow samples, and samples from these sites were also positive for one or more sewage-associated marker genes. The results presented in this study could provide valuable information to the water quality managers for mitigation of emerging contaminants and sewage pollution in urban rivers or similar aquatic environments.

There are several limitations of this study. For example, the grab sampling method used in this study only provided a snapshot of microbial targets in the studied river system. Future studies would also benefit from using flow-weighted composite samples, particularly during stormflow conditions, to characterize *E. coli*, ARGs, and sewage-associated marker abundances. We used a moderate volume (i.e., 500 mL) of water samples for the concentration of microbial targets, however, during the baseflow, processing a large volume of water samples may increase the detection sensitivity. In this study, for river water samples, left-censored data analysis techniques were used to assess differences in microbial target abundance by areas and flow conditions, as well as to characterize the correlation between ARGs, *E. coli*, and sewage-markers because several microbial targets were not always detected, and/or detected in abundances too low to quantify (Helsel, 2011). While baseflow conditions were sampled on three occasions, stormflow conditions (i.e., 57 mm rainfall) were sampled on only one occasion because the study area did not receive any rainfall except for only once during the study period. Since the power of statistical analyses decreases with increased data censoring and unbalanced sampling, future studies are needed to confirm the findings presented here.

Multivariate statistical analyses appropriate for left-censored data were used in this study to understand the correlation between fecal indicator microorganisms and ARGs in untreated sewage and river water, as well as to test hypotheses about land use and flow conditions with respect to ARGs and fecal indicator microorganisms in samples collected from the Brisbane River system. The abundance of ARGs and sewage-associated markers increased during stormflow conditions. Specifically, HF183 and crAssphage may serve as potential indicators of ARGs of sewage origin that merit further investigation. It can be difficult to identify cause-effect relationships between ARGs and sewage-associated markers in an uncontrolled, monitoring-based study. However, this study demonstrated that sewage-associated marker genes can be used to indicate the presence of the group of sewage-associated ARGs analyzed in this study under stormflow conditions. Data presented in this study may be valuable to water quality managers to manage sewage pollution and ARGs or pathogens in environmental waters. Future work, particularly mesocosm experiments, can build on this study to better understand the relationship between ARGs, HF183, and crAssphage genome.

## Data Availability Statement

The raw data supporting the conclusions of this article will be made available by the authors, without undue reservation.

## Author Contributions

WA: investigation, resources, and writing—original draft. PG: sampling and study design. KH: sample analysis and writing—original draft. SJ: sample analysis and writing—original draft. DA: sampling and data collection. ED: writing—original draft. SS: resources and writing—original draft. ES: statistical analysis and writing—original draft. All authors listed have made a substantial, direct and intellectual contribution to the work, and approved it for publication.

## Conflict of Interest

The authors declare that the research was conducted in the absence of any commercial or financial relationships that could be construed as a potential conflict of interest.
