# Supplementary material for: Antibiotic Resistance and Sewage-Associated Marker Genes in Untreated Sewage and a River Characterized During Baseflow and Stormflow
Source: Front Microbiol. 2021 Jun 11;12:632850. doi: 10.3389/fmicb.2021.632850 (PMC8226142; doi:10.3389/fmicb.2021.632850)
Supplement: Supplementary file 1 [file Data_Sheet_1.DOCX]

**Supplementary materials**

**Supplementary Table ST1:** Primer, probes and qPCR cycling parameters used in this study.

| Assays | Primers and probes | Base pairs | Primer and probe concentration (nM) | Cycling parameters | References |
| --- | --- | --- | --- | --- | --- |
| Sketa22 | F: GGTTTCCGCAGCTGGG  R: CCGAGCCGTCCTGGTCTA  P: FAM-AGTCGCAGGCGGCCACCGT-TAMRA | 77 | 500  500  400 | 10 min at 95°C, 40 cycles of 15 s at 95°C, 45 s at 63°C | Haugland et al. 2005 |
| *E. coli* 23S rRNA gene | F: GGT AGA GCA CTG TTT TGG CA  R: TGT CTC CCG TGA TAA CTT TCTC  P: FAM- TCA TCC CGA CTT ACC AAC CCG -TAMRA | 100 | 800  800  80 | 10 min at 95°C, 40 cycles of 15 s at 95°C, 60 s at 60°C | Chern et al. 2011 |
| *aacA* | F: GTGTAACACGCAAGCACGAT  R: AGCCTCCGCGATTTCATAC | 197 | 300  300 | 10 min at 95°C, 40 cycles of 15 s at 95°C, 60 s at 63°C | Szczepanowski et al. 2009 |
| *bla*_ctx-m-32_ | F: CGTCACGCTGTTGTTAGGAA  R: CGCTCATCAGCACGATAAAG | 156 | 300  300 | 10 min at 95°C, 40 cycles of 15 s at 95°C, 60 s at 60°C | Szczepanowski et al. 2009 |
| *bla*_KPC_ | F: GATACCACGTTCCGTCTGG  R: GCAGGTTCCGGTTTTGTCTC | 252 | 300  300 | 10 min at 95°C, 40 cycles of 15 s at 95°C, 60 s at 60°C | Hindiyeh et al. 2008 |
| *bla*_VIM_ | F: CGCAGCTTTCTGGTTGGTAT  R: CGTGTCACCGAGTTTCTGAG | 180 | 300  300 | 10 min at 95°C, 40 cycles of 15 s at 95°C, 60 s at 60°C | Szczepanowski et al. 2009 |
| *ermF* | F: CGACACAGCTTTGGTTGAAC  R: GGACCTACCTCATAGACAAG | 309 | 300  300 | 10 min at 95°C, 40 cycles of 15 s at 95°C, 60 s at 58°C | Ma et al. 2011 |
| *intl1* | F: CCTCCCGCACGATGATC  R: TCCACGCATCGTCAGGC | 280 | 300  300 | 10 min at 95°C, 40 cycles of 15 s at 95°C, 60 s at 60°C | Goldstein et al. 2001 |
| *sul1* | F: CCGTTGGCCTTCCTGTAAAG  R: TTGCCGATCGCGTGAAGT | 67 | 300  300 | 10 min at 95°C, 40 cycles of 15 s at 95°C, 60 s at 55°C | Heuer et al. 2007 |
| *sul2* | F: GACAGTTATCAACCCGCGAC  R: GTCTTGCACCGAATGCATAA | 147 | 300  300 | 10 min at 95°C, 40 cycles of 15 s at 95°C, 60 s at 60°C | Szczepanowski et al. 2009 |
| *tet(M)* | F: GTGGACAAAGGTACAACGAG  R: CGGTAAAGTTCGTCACACAC | 406 | 300  300 | 10 min at 95°C, 40 cycles of 15 s at 95°C, 60 s at 59°C | Ng et al. 2001 |
| *vanA* | F: GTAGGCTGCGATATTCAAAGC  R: CGATTCAATTGCGTAGTCCAA | 231 | 300  300 | 10 min at 95°C, 40 cycles of 15 s at 95°C, 60 s at 60°C | Bell et al. 1998 |
| HF183 | F: ATCATGAGTTCACATGTCCG  R: CTTCCTCTCAGAACCCCTATCC  P: FAM-CTAATGGAACGCATCCC-MGB | 173 | 1000  1000  100 | 10 min at 95°C, 40 cycles of 15 s at 95°C, 60 s at 60°C | Green et al. 2014 |
| Lachno3 | F: CAACGCGAAGAACCTTACCAAA  R: CCCAGAGTGCCCACCTTAAAT  P: FAM-CTCTGACCGGTCTTTAATCGGA-TAMRA | 187 | 1000  1000  100 | 10 min at 95°C, 40 cycles of 15 s at 95°C, 60 s at 60°C | Feng et al. 2018 |
| CrAssphage | F: CAGAAGTACAAACTCCTAAAAAACGTAGAG  R: GATGACCAATAAACAAGCCATTAGC  P: FAM-AATAACGATTTACGTGATGTAAC-BHQ1 | 125 | 1000  1000  100 | 10 min at 95°C, 40 cycles of 15 s at 95°C, 60 s at 60°C | Stachler et al. 2017 |

**Supplementary Table ST2:** Prevalence of *E. coli*, ARGs and sewage-associated marker genes in water samples collected during the baseflow and stormflow from Brisbane River system.

| Events | Sites | EC 23S rRNA gene | *aacA* | *bla_kpc_* | *bla*_VIM_ | *bla_ctx-m-3_*_2_ | *ermF* | *intl1* | *sul1* | *sul2* | *tet(*M) | *vanA* | HF183 | Lachno3 | CrAssphage |
| --- | --- | --- | --- | --- | --- | --- | --- | --- | --- | --- | --- | --- | --- | --- | --- |
| Event 1 (baseflow) | BR1 | + |  | + |  |  |  |  |  |  |  |  |  |  |  |
|  | BR2 | + |  | + |  |  |  | + | + |  |  |  |  |  |  |
|  | BR3 | + |  | + |  |  |  | + | + |  |  |  |  |  |  |
|  | BR4 | + |  |  |  |  |  |  |  |  |  |  |  |  |  |
|  | BR5 | + |  |  |  |  |  |  |  |  |  |  |  |  |  |
|  | OX1 | + |  | + |  |  |  | + | + |  |  |  |  |  |  |
|  | BR6 | + |  |  |  |  |  |  |  |  |  |  |  |  |  |
|  | BR7 | + |  |  |  |  |  |  |  |  | + |  |  |  |  |
|  | BR8 | + |  |  |  |  |  |  |  |  |  |  |  |  |  |
|  | BR9 | + |  |  |  |  |  |  |  |  |  |  |  |  |  |
|  | BR10 | + |  |  |  |  |  |  |  |  |  |  |  |  |  |
|  | BR11 | + |  |  |  |  |  |  |  |  |  |  |  |  |  |
|  | BR12 | + |  |  |  |  |  |  |  |  | + |  |  |  |  |
|  | BC1 | + |  |  |  |  |  |  |  |  |  |  |  |  |  |
| Event 2 (baseflow) | BR1 | + |  | + |  |  |  | + | + |  |  |  |  |  |  |
|  | BR2 | + |  | + |  |  |  | + | + |  |  |  |  |  |  |
|  | BR3 | + |  |  |  |  |  | + |  |  | + |  |  |  |  |
|  | BR4 | + |  |  |  |  |  |  |  |  | + |  |  |  |  |
|  | BR5 | + |  |  |  |  |  |  |  |  |  |  |  |  |  |
|  | OX1 | + |  | + |  |  | + | + |  |  | + |  |  |  |  |
|  | BR6 | + |  |  |  |  |  |  |  |  |  |  |  |  |  |
|  | BR7 | + |  |  |  |  |  |  |  |  |  |  |  |  |  |
|  | BR8 | + |  | + |  |  |  | + | + |  | + |  | + | + | + |
|  | BR9 | + |  |  |  |  |  |  |  |  |  |  |  |  |  |
|  | BR10 | + |  |  |  |  |  |  |  |  |  |  |  |  |  |
|  | BR11 | + |  |  |  |  |  |  |  |  |  |  |  |  |  |
|  | BR12 | + |  |  |  |  |  |  |  |  |  |  |  |  |  |
|  | BC1 | + |  |  |  |  |  |  |  |  |  |  |  |  |  |
| Event 3 (baseflow) | BR1 | + |  | + |  |  |  |  | + |  |  |  |  |  |  |
|  | BR2 | + |  | + |  |  |  | + | + |  |  |  |  |  |  |
|  | BR3 | + | + |  |  |  |  | + | + |  |  |  |  |  |  |
|  | BR4 | + |  |  |  |  |  |  |  |  |  |  |  |  |  |
|  | BR5 | + |  |  |  |  |  |  |  |  |  |  |  |  |  |
|  | OX1 | + |  | + |  |  | + | + | + |  |  |  |  |  |  |
|  | BR6 | + |  |  |  |  |  |  |  |  |  |  |  |  |  |
|  | BR7 | + |  |  |  |  |  |  |  |  |  |  |  |  |  |
|  | BR8 | + |  |  |  |  |  |  |  |  |  |  |  |  |  |
|  | BR9 | + |  |  |  |  |  |  |  |  |  |  |  |  |  |
|  | BR10 | + |  |  |  |  |  |  |  |  |  |  |  |  |  |
|  | BR11 | + |  |  |  |  |  |  |  |  |  |  |  |  |  |
|  | BR12 | + |  |  |  |  |  |  |  |  |  |  |  |  |  |
|  | BC1 | + |  |  |  |  |  |  |  |  |  |  |  |  |  |
| Event 4 (stormflow) | BR1 | + |  | + | + |  | + | + | + | + |  |  |  |  |  |
|  | BR2 | + | + | + | + |  |  | + | + | + |  |  | + |  | + |
|  | BR3 | + |  |  |  |  |  | + | + | + |  |  |  |  |  |
|  | BR4 | + |  |  |  |  |  | + | + |  |  |  |  | + |  |
|  | BR5 | + |  |  |  |  | + | + | + | + |  |  | + | + | + |
|  | OX1 | + |  |  |  |  | + | + | + | + |  |  | + | + | + |
|  | BR6 | + |  |  |  |  | + |  |  |  |  |  | + |  | + |
|  | BR7 | + |  |  |  |  |  |  |  |  | + |  |  |  |  |
|  | BR8 | + |  |  |  |  | + |  |  |  | + |  | + |  | + |
|  | BR9 | + |  |  |  |  |  |  |  |  |  |  |  |  |  |
|  | BR10 | + |  |  |  |  |  |  |  |  |  |  |  |  |  |
|  | BR11 | + |  |  |  |  |  |  |  |  |  |  |  |  |  |
|  | BR12 | + |  |  |  |  | + | + | + | + | + |  | + | + | + |
|  | BC1 | + | + | + | + | + | + | + | + | + | + |  | + | + | + |

**References**

1. Bell JM, Paton JC, Turnidge J (1998) Emergece of vancomycin-resistant enterococci in Australia: Phenotypic and genotypic characteristics of isolates. J Clin Microbiol. 36:2187-2190.
2. Chern EC, Siefring S, Paar J Doolittle M, Haugland RA (2011) Comparison of quantitative PCR assays for *Escherichia coli* targeting ribosomal RNA and single copy genes. Lett Appl Microbiol. 52:298−306.
3. Feng S, Bootsma M, McLellan SL (2018) Novel human-associated *Lachnospiraceae* genetic markers improve detection of fecal pollution sources in urban waters. Appl. Environ. Microbiol. 84:pii: e00309-18.
4. Goldstein C, Lee MD, Sanchez S, Hudson C, Phillips B, Register B, Grady M, Liebert C, Summers AO, White DG, Maurer JJ (2001) Incidence of class 1 and 2 integrases in clinical and commensal bacteria from livestock, companion animals, and exotics. Antimicrob. Agents Chemo. 45:723-726.
5. Green HC, Haugland RA, Varma M, Millen HT, Borchardt MA, Field KG, Walters WA, Sivaganesan M, Kelty CA, Shanks OC. 2014. Improved HF183 quantitative real-time PCR assay for characterization of human fecal pollution in ambient surface water samples. Appl Environ Microbiol. 80:3086-3094.
6. Haugland RA, Siefring SC, Wymer LJ, Brenner KP, Dufour AP (2005) Comparison of Enterococcus measurements in freshwater at two recreational beaches by quantitative polymerase chain reaction and membrane filter culture analysis. Water Res. 39:559-568.
7. Heuer H, Smalla K (2007) Manure and sulfadiazine synergistically increased bacterial antibiotic resistance in soil over at least two months. Environ. Microbiol. 9:657-666.
8. Hindiyeh M, Smollen G, Grossman Z (2008) Rapid detection of *bla*_KPC_ carbapenemase genes by real-time PCR. J. Clin. Microbiol. 46:2879-2883.
9. Ma Y, Wilson CA, Novak JT, Riffat R, Aynur S, Murthy S, Pruden A (2011) Effect of various sludge digestion conditions on sulfonamide, macrolide, and tetracycline resistance genes and class I integrons. Environ. Sci. Technol. 2011, 45, 7855-7861.
10. Ng L-K, Martin I, Alfa M, Mulvey M (2001) Multiplex PCR for the detection of tetracycline resistant genes. Mol. Cell Prob. 15:209-215.
11. Stachler E, Kelty C, Sivaganesan M, Li X, Bibby K, Shanks OC. 2017. Quantitative CrAssphage PCR assays for human fecal pollution measurement. Environ. Sci. Technol. 51:9146-9154.
12. Szczepanowski R, Linke B, Krahn I (2009) Detection of 140 clinically relevant antibiotic-resistance genes in the plasmid metagenome of wastewater treatment plant bacteria showing reduced susceptibility to selected antibiotics. Microbiol. 155:2306–2319.
