# Supplementary figures and images for: Antibiotic Resistance and Sewage-Associated Marker Genes in Untreated Sewage and a River Characterized During Baseflow and Stormflow
Source: Front Microbiol. 2021 Jun 11;12:632850. doi: 10.3389/fmicb.2021.632850 (PMC8226142; doi:10.3389/fmicb.2021.632850)

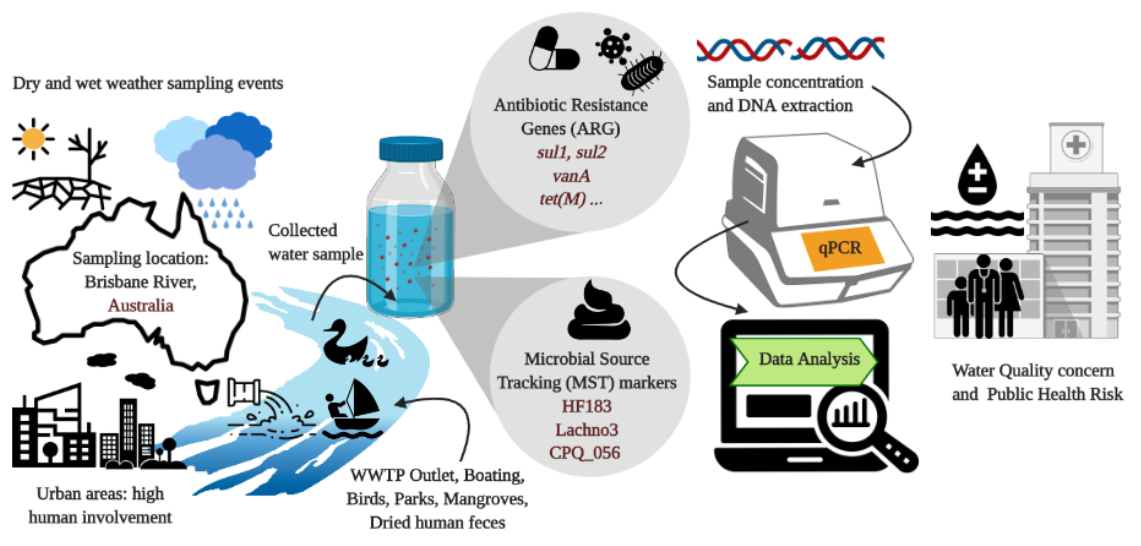

Supplement: Supplementary file 2 [file Image_1.PNG]
